# The Effects of Heat Treatment on the Microstructure and Mechanical Properties of a Selective Laser Melted AlCoFeNi Medium-Entropy Alloy

**DOI:** 10.3390/ma17071582

**Published:** 2024-03-29

**Authors:** Xinyang Han, Xiangwei Li, Bokai Liao, Youzhao Zhang, Lei Xu, Xingpeng Guo, Shuyan Zhang

**Affiliations:** 1 School of Physics and Materials Science, Guangzhou University, Guangzhou 510006, China; 2 Centre of Excellence for Advanced Materials, Dongguan 523808, China; 3 School of Chemistry and Chemical Engineering, Guangzhou University, Guangzhou 510006, China; 4 Robotics and Artificial Intelligence Division, Hong Kong Productivity Council, Kowloon, Hong Kong SAR 999077, China

**Keywords:** medium-entropy alloy, AlCoFeNi, heat treatment, selective laser melting, microstructure, mechanical properties

## Abstract

A single body-centered cubic (BCC)-structured AlCoFeNi medium-entropy alloy (MEA) was prepared by the selective laser melting (SLM) technique. The hardness of the as-built sample was around 32.5 HRC. The ultimate tensile strength (UTS) was around 1211 MPa, the yield strength (YS) was around 1023 MPa, and the elongation (El) was around 10.8%. A novel BCC + B2 + face-centered cubic (FCC) structure was formed after aging. With an increase in aging temperature and duration, the number of fine grains increased, and more precipitates were observed. After aging at 450 °C for 4 h, the formed complex polyphase structure significantly improved the mechanical properties. Its hardness, UTS, YS, and El were around 45.7 HRC, 1535 MPa, 1489 MPa, and 8.5%, respectively. The improvement in mechanical properties was mainly due to Hall–Petch strengthening, which was caused by fine grains, and precipitation strengthening, which was caused by an increase in precipitates after aging. Meanwhile, the FCC precipitates made the alloy have good toughness. The complex interaction of multiple strengthening mechanisms leads to a good combination of strength, hardness, and toughness.

## 1. Introduction

Medium-entropy alloys (MEAs), which consist of three to four elements, are derived from the concept of high-entropy alloys (HEAs) and demonstrate a mixing entropy ranging from 1 R to 1.5 R (where R represents the gas constant). Similar to HEAs, MEAs demonstrate superior mechanical properties, exceptional high-temperature stability, and improved corrosion resistance compared to conventional alloys [1]. Great attention has been paid to MEAs because of their excellent mechanical properties [2,3]. Laplanche et al. studied the equiatomic CoCrNi MEA produced by vacuum induction melting, which exhibited a single face-centered cubic (FCC) structure. This MEA exhibited an ultimate tensile strength (UTS) of 870 MPa, a yield strength (YS) of 360 MPa, and an elongation (El) of 38% at 293 K [2]. Moravcik et al. studied the equiatomic CoCrNi MEA through mechanical alloying (MA) followed by spark plasma sintering (SPS). It exhibited a dual-phase, FCC (94.4%), and body-centered cubic (BCC) (5.6%) structure. The resulted UTS was 1024 MPa, and the El was 26% [4]. Non-equiatomic CoFeNi MEAs were prepared by Paganotti et al. using arc melting. These arc-melted MEAs exhibited high UTS and good plasticity [5].

Due to the high mixing entropy of HEAs and MEAs, single-phase solid solutions (FCC or BCC phases) are usually formed [6,7]. However, a single-phase FCC or BCC alloy usually exhibits low hardness or toughness [8]. Li et al. fabricated a single FCC-structured CoFeNi MEA by vacuum arc melting. The UTS was 495.2 MPa, the YS was 198.6 MPa, and the hardness was 141.1 HV [9]. To enhance the mechanical properties of single-phase-structured MEAs, numerous studies have been conducted on the addition of Al to MEAs composed of Fe, Co, Ni, Cr, and other transition elements, which can create a complex phase structure and leads to smaller grain sizes, thereby improving the mechanical properties [10]. Dasari et al. reported that the addition of Al to an FCC matrix composed of Cr, Co, Fe, and Ni elements effectively induced an ordering trend, leading to the formation of a B2/L1_2_ phase [11,12,13]. With an increasing Al content, the precipitates in the alloy gradually increased, and the mechanical properties of the alloys gradually increased [8,14]. Most of the above studies used MEAs with an equal atomic ratio and an FCC structure; however, there is limited research on improving BCC-matrix FeCoNi MEAs. In this paper, a non-equiatomic AlCoFeNi MEA with a BCC-structured matrix was designed by changing the content of Co and adding a small amount of Al.

Compared to the traditional manufacturing methods for MEAs, such as vacuum melting and casting, the higher cooling rate of the additive manufacturing (AM) process helps to reduce segregation and produces finer grains [15,16]. This makes AM a new avenue for manufacturing high-performance alloys, which has attracted significant attention. Selective laser melting (SLM), based on powder bed fusion, has been recognized as a promising AM technology [17,18]. For example, a Cr_10_Co_15_Fe_60_Ni_15_ alloy, prepared by SLM, exhibited significantly enhanced strength and ductility compared with its cast counterpart [19]. Han et al. fabricated a near fully dense hierarchical equimolar CrCoNi MEA via SLM. A YS of 651 MPa, a UTS of 907.7 MPa, and an El of 35.8% at 293 K was obtained, which was much higher than those achieved by traditional alloy production methods [20]. An equimolar CoCrNi MEA with a single FCC phase was prepared via SLM, whose mechanical properties were twice as those of a cast or wrought CoCrNi MEA [21]. Due to the good thermal conductivity of the CoFeNi MEA, an additive manufactured CoFeNi MEA has been widely used in conformal cooling molds [22].

In addition to pre-treatment during the manufacturing of alloys, post-treatment is also essential for good performance. Aging is known to be an effective method for improving the strength and hardness of precipitation-hardened alloys. Proper aging can optimize the distribution and morphology of precipitates, therefore achieving a better balance between strength and toughness. A phase transformation pathway was designed by Dasari et al. for an equiatomic Al_0.3_CoFeNi MEA. The homogenized and cold-rolled sample was two-step annealed at 700 °C and 600 °C, respectively, for 50 h, resulting in a novel multi-scale microstructure. This microstructure consisted of fine-scale FCC + L1_2_ grains interspersed with B2 + BCC grains. This resulted in a YS of around 1490 MPa, a UTS of around 1663 MPa, and an El of around 12% at room temperature [23]. Gwalani et al. studied the tensile strength of an Al_0.3_CoCrFeNi HEA. When the solution-treated and cold-rolled sample was annealed at 550 °C for 24 h, the UTS reached 1850 MPa, which was significantly higher than the sample without annealing treatment (with a UTS of around 400 MPa) [24]. Song et al. studied the effect of heat treatment on the mechanical behaviors of SLM 18Ni-300. After being aged at 490 °C for 2 h, the YS and the UTS were around 1775 MPa and 1820 MPa, respectively, which were much higher than those of the as-built sample (the YS was around 928 MPa, and the UTS was around 1016 MPa) [25].

In this study, a single BCC-structured FeCoNiAl MEA was prepared by the SLM technique. The effects of heat treatment on the microstructure and mechanical properties were investigated.

## 2. Materials and Experimental Methods

### 2.1. Powder Characteristics

**Figure 1:**
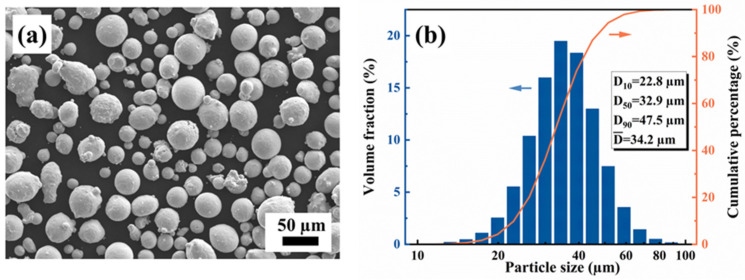
(**a**) SEM morphology and (**b**) particle size distribution of the powder.

A gas-atomized non-equiatomic AlCoFeNi MEA powder was employed in the present study. A scanning electron microscope (SEM, JSM-IT1800, JEOL Ltd., Tokyo, Japan) was employed to observe the powder morphology, and the results are shown in Figure 1a. Most of the powder exhibits a uniform diameter and spherical shape, while some parts of the powder have satellites attached to their surfaces. There is also a little amount of powder showing an irregular shape. The size of the powder was measured by a laser particle-sized analyzer (HORIBA LA-350, HORIBA, Osaka, Japan). Figure 1b shows the size of the distribution of the powder. The maximum particle size reaches up to 100 μm, and a majority of the powder falls within the range of 20 to 60 μm.

### 2.2. SLM Process

A metallic selective laser melting machine (HB-150, Guangdong Hanbang3D Technology Co., Ltd., Zhongshan, China) equipped with a 500 W single-mode fiber laser and a laser spot diameter of 30 μm was used to manufacture samples on a 304 stainless steel substrate. To minimize the residual stress in the samples and prevent deformation and cracking, the substrate was preheated to 100 °C before printing [26]. Moreover, to protect the alloy from oxidation, dust, and splashes, the printing procedure was carried out in an argon atmosphere [27].

**Figure 2:**
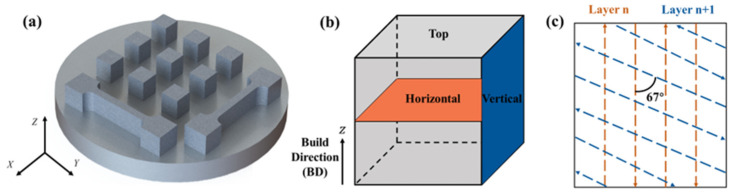
Schematic diagram of (**a**) the printed cubic samples and tensile samples; (**b**) selected direction of test samples; and (**c**) laser scanning strategy.

**Figure 3:**
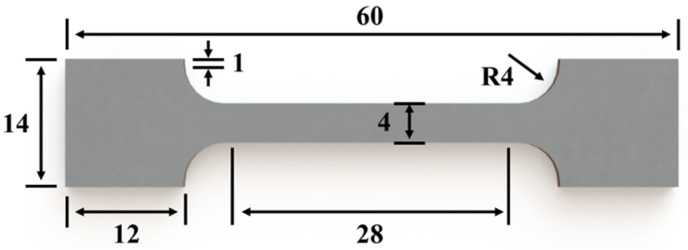
Diagrammatic sketch of tensile specimen (unit: mm).

The density of the samples manufactured by various laser scanning speeds and laser power settings was tested to optimize the printing parameters. Finally, a laser scanning speed of 700 mm/s and a laser power of 200 W were selected. A scanning spacing of 100 μm and a layer thickness of 30 μm were set. The laser energy density was calculated to be 95.24 J/mm^3^. As shown in Figure 2a, cube samples (10 × 10 × 10 mm^3^) and tensile samples (the shape is shown in Figure 3, and the height was 20 mm) were printed. Figure 2b shows the build direction (BD) of the samples. The vertical direction of the sample was chosen to observe the molten pool, while the horizontal direction was selected for the molten channel, microstructure observation, and tests on the mechanical properties. In the present experiment, the vertical direction was aligned parallel to the build direction, while the horizontal direction was perpendicular to the build direction. Additionally, Figure 2c illustrates the S-shaped trip scanning strategy employed by the laser during the trip, with the direction of the arrow representing the movement of the laser. Furthermore, to minimize anisotropy in the X–Y plane, a scanning rotational direction of 67° was implemented for every two adjacent layers [28,29].

### 2.3. Microstructure Characterization and Analysis

After sectioning and polishing, an optical microscope (OM, Axio Scope 5, Zeiss, Chiyoda City, Japan) was used to capture OM images, and the relative density was calculated using the Image J 1.53t software. Subsequently, the morphology of the molten channel and the molten pool was observed after being etched with a reagent (5 g of FeCl_3_, 50 mL of HCl, and 100 mL of H_2_O).

The phase identification of the samples was performed using X-ray diffraction (XRD, Ultima VI, Rigaku, Tokyo, Japan) under Cu Kα radiation (40 mA, 40 kV) with a scanning step of 0.02° and of 4°/min and a scanning angle from 20° to 90°. The OM and SEM were employed for the microstructure observation of the etched samples. The samples underwent vibration polishing for four hours, and the grains and crystal orientation were investigated using an electron backscatter diffraction (EBSD, JSM-IT1800, Clevedon, UK) with a scanning step of 0.055 μm. The precipitate characterization was carried out using a transmission electron microscope (TEM, FEI Tecnai G2 F30, FEI, Hillsboro, OR, USA) operating at 300 kV, and a dispersive X-ray spectrometry (EDS) system was also performed to further examine the precipitate composition. Additionally, the fracture morphology of the tensile samples was examined using SEM.

### 2.4. Heat Treatment

The as-built block samples were machined into small pieces with a thickness of about 3 mm prior to heat treatment. To investigate the influence of temperature and duration on the microstructure and mechanical properties, some samples underwent aging treatments at different temperatures of 410 °C and 430 °C for 2 h; and 450 °C for 2 h, 4 h, and 6 h, respectively, according to previous studies [30,31]. All samples were air-cooled. Table 1 presents the details of the heat treatment conditions, namely conditions B to F. Condition A represents the as-built sample without heat treatment, serving as the reference samples. To prevent oxidation during the heat treatment, tensile samples were hermetically sealed in vacuum silicon tubes.

### 2.5. Mechanical Test

The hardness of the polished samples was determined using a Rockwell hardness tester (Rockwell 574, Rockwell Automation Inc., Milwaukee, WI, USA) at a test force of 10 kgf and a hold for 2 s. Four points were measured on each sample, and the average value was used to represent the sample’s hardness. The tensile samples were prepared according to the diagram shown in Figure 3. These samples were cut from the large tensile samples depicted in Figure 2a into sheets with a thickness of approximately 2 mm. The tensile tests were conducted at room temperature using a fatigue testing system (Instron 8802, Instron, Norwood, MA, USA) equipped with an extensometer and an initial strain rate of 1 mm/min. Two samples of each condition were tested to ensure the accuracy and reproducibility of the results.

## 3. Results

### 3.1. Microstructure of the As-Built AlCoFeNi MEA

**Figure 4:**
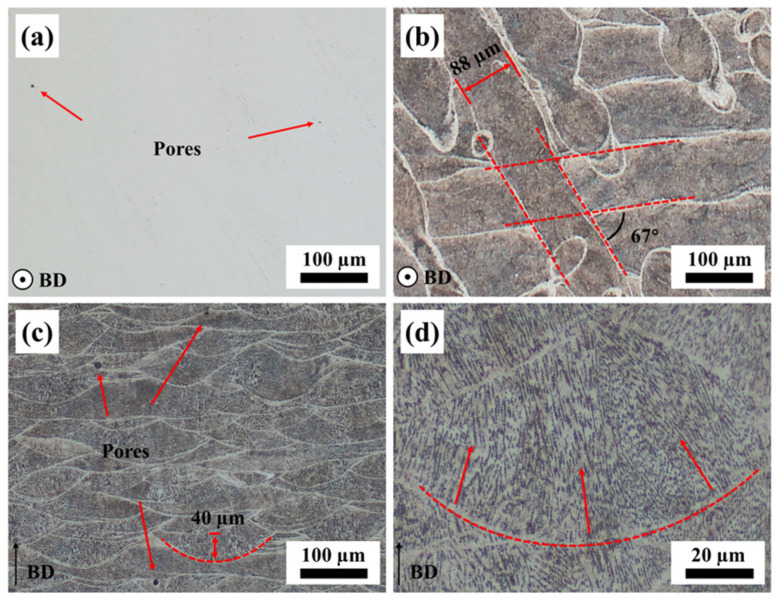
OM images of the as-built AlCoFeNi MEA: (**a**) polished and (**b**) etched horizontal section, (**c**) etched vertical section, and (**d**) enlarged image of a single molten pool. BD represents the build direction.

Figure 4 presents the OM images of the as-built AlCoFeNi MEA at a relative density of 99.97%, which was measured using the Image J software. Only a few pores were present in the horizontal section, and almost no cracks could be observed (Figure 4a). After etching, a slender structure with a width of approximately 88 μm was observed (Figure 4b), which represented the laser solidification track. It was also evident that the rotation angle of the laser between adjacent layers was 67°, and the adjacent solidification track had a positive metallurgical effect. The circles in the horizontal OM images were retained due to the uneven polish. In the vertical section (Figure 4c,d), the boundary of the molten pool exhibits a regular semi-elliptical shape with a fish-scale distribution pattern of approximately 40 μm in height and around 170 μm in width. Columnar grains perpendicular to the molten pool boundary were observed due to the temperature gradient. Overall, the metallurgical bond between the layers was satisfactory, and the molten pool exhibited a few pores that were inevitably formed.

**Figure 5:**
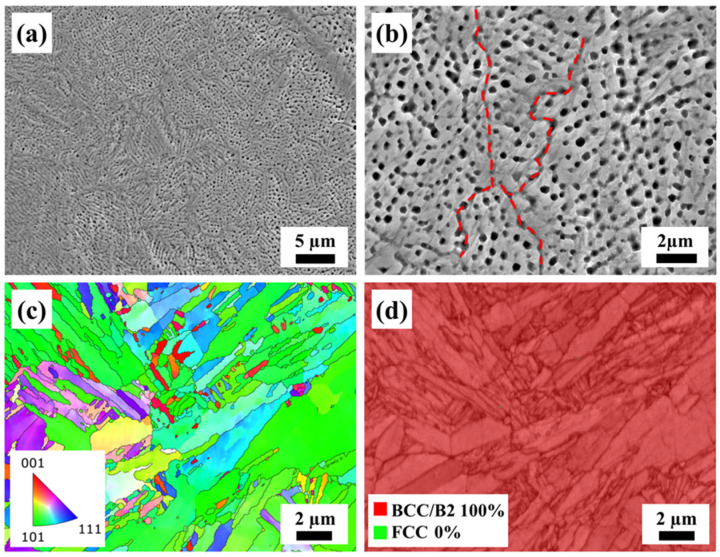
Characterization of the as-built AlCoFeNi MEA: (**a**) SEM micrographs and (**b**) magnified image of (**a**), (**c**) inverse pole figure map, and (**d**) the corresponding phase distribution of (**c**).

Figure 5 presents the SEM and EBSD micrographs of the as-built AlCoFeNi MEA. As depicted in Figure 5a, heterogeneous cellular structures with sinuous boundaries were evident within the grains, which are typical structures of SLMs [21,32]. The red dashed lines in Figure 5b represent grain boundaries. Moreover, no noticeable precipitates were observed within the structure. As can be seen from Figure 5c, the grain orientation had a certain preferred orientation in the <101> direction. The grain size was not uniform in the as-built samples, which contained both large grains of over 10 μm in length and nano-sized fine grains. It can be seen from Figure 5d that almost no FCC phase could be detected in the as-built samples.

### 3.2. Microstructure of the Heat-Treated AlCoFeNi MEA

**Figure 6:**
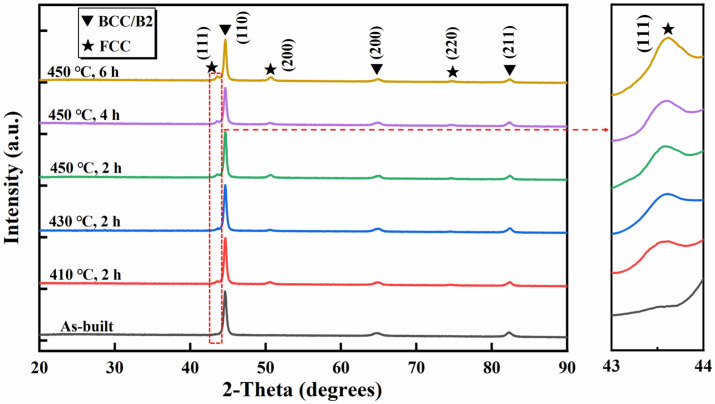
XRD spectra of the as-built and heat-treated AlCoFeNi MEA.

Figure 6 presents the XRD spectra of the samples after different heat treatments. The as-built samples exhibited a single BCC structure. After heat treatment, phase transformation occurred, resulting in the formation of an FCC phase in the matrix. The Jade 6.5 software was used to calculate the fraction of the FCC in the specimens subjected to different aging conditions. After aging for 2 h at 410 °C, 430 °C, and 450 °C, the fraction of the FCC was 5.4%, 6.3%, and 8.2%, respectively. And the fraction of the FCC was 8.5% and 11.9% after aging at 450 °C for 4 h and 6 h, respectively. As shown in the enlarged image of the XRD spectra, the peaks of the FCC phase were slightly shifted with the increase in aging temperature and duration. It was difficult to distinguish the specific ordered B2 phase or disordered BCC phase structure due to limitations of the XRD spectra. However, we can quickly determine the matrix and FCC precipitates through analyzed XRD spectra. This allows us to compare the experimental data with the results of previous studies.

**Figure 7:**
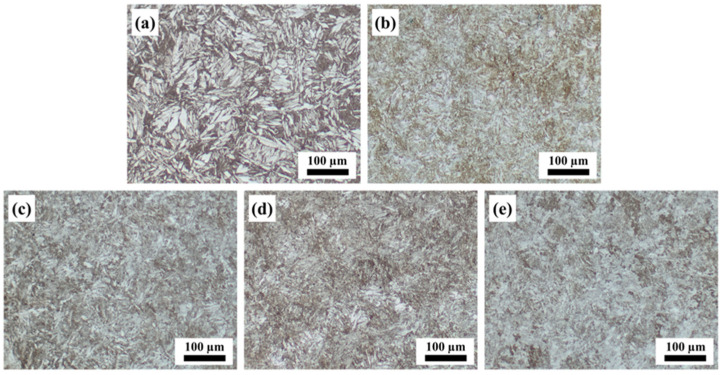
OM images of the aged AlCoFeNi MEA: (**a**) 410 °C for 2 h, (**b**) 430 °C for 2 h, (**c**) 450 °C for 2 h, (**d**) 450 °C for 4 h, and (**e**) 450 °C for 6 h.

Figure 7 presents the OM images of the heat-treated samples. As shown in Figure 7a–c, the aging temperature had a significant effect on the microstructures of the AlCoFeNi MEA. After 2 h of aging at 410 °C, numerous unevenly distributed martensitic structures could be observed. And these martensitic structures were coarse, ranging in length from 30 to 80 μm. However, after being aged for 2 h at 430 °C and 450 °C, the martensitic structures were significantly refined, as shown in Figure 7b,c. Some acicular and columnar martensites could be found in the matrix. The size of the martensitic structures gradually decreased to less than 20 μm in the samples aged at 450 °C for 2 h. With an extension of the aging time from 2 h to 6 h at 450 °C, it can be seen that the martensitic structures were refined but not obviously (Figure 7c–e).

**Figure 8:**
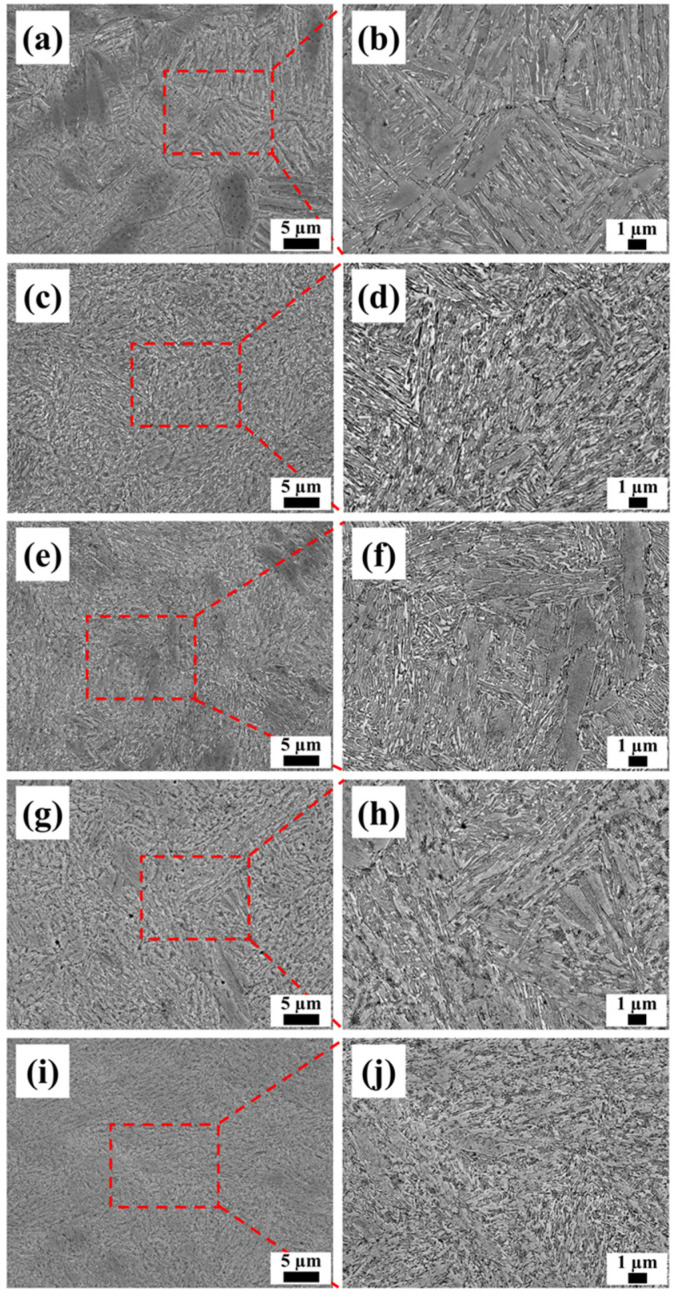
SEM micrographs of the aged AlCoFeNi MEA: (**a**,**b**) 410 °C for 2 h; (**c**,**d**) 430 °C for 2 h; 450 °C for (**e**,**f**) 2 h, (**g**,**h**), 4 h, and (**i**,**j**) 6 h.

Figure 8 shows the SEM images of the heat-treated samples. Compared to the as-built samples in Figure 5a, numerous discontinuous granular and acicular precipitates, ranging in length from 0.1 μm to 2 μm, were observed within grains and grain boundaries in the heat-treated samples. At an elevated temperature and a longer duration, the amount of precipitates increased, and the precipitates gradually grew, and the original cellular crystal structures gradually disappeared. These results indicate that an aging treatment could effectively promote the precipitation in AlCoFeNi MEAs, and the distribution of precipitates become more uniform with an increase in the aging temperature and duration.

**Figure 9:**
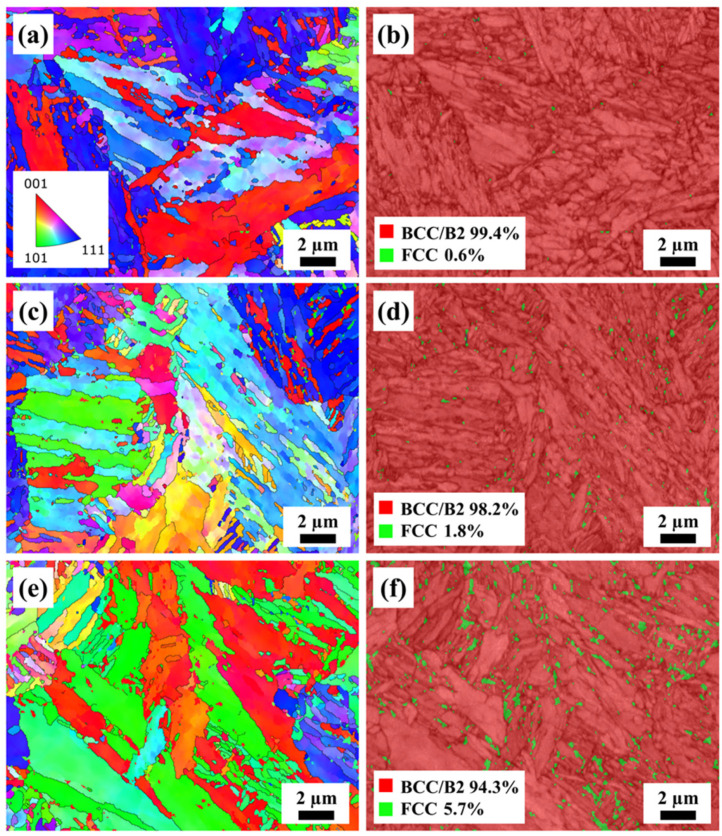
Inverse pole figure (IPF) maps of the aged AlCoFeNi MEA: (**a**) 410 °C for 2 h, (**c**) 450 °C for 2 h and (**e**) 6 h. (**b**), (**d**), and (**f**) are the phase maps of (**a**), (**c**), and (**e**), respectively.

Figure 9 presents the EBSD micrographs of representative aged samples. Compared with Figure 5c, more nano-sized precipitates within the grains and grain boundaries can be seen. And it can be observed that the number of nano-sized precipitates was significantly increased with the increase in aging temperature and duration. As shown in Figure 9b,d,f, the amount of the FCC phase increased, and the FCC phase gradually grew. The volume fraction of the FCC phase increased from 0.6% to 1.8% with an increase in temperature from 410 °C to 450 °C for 2 h and then increased to 5.7% when the aging duration increased to 6 h.

**Figure 10:**
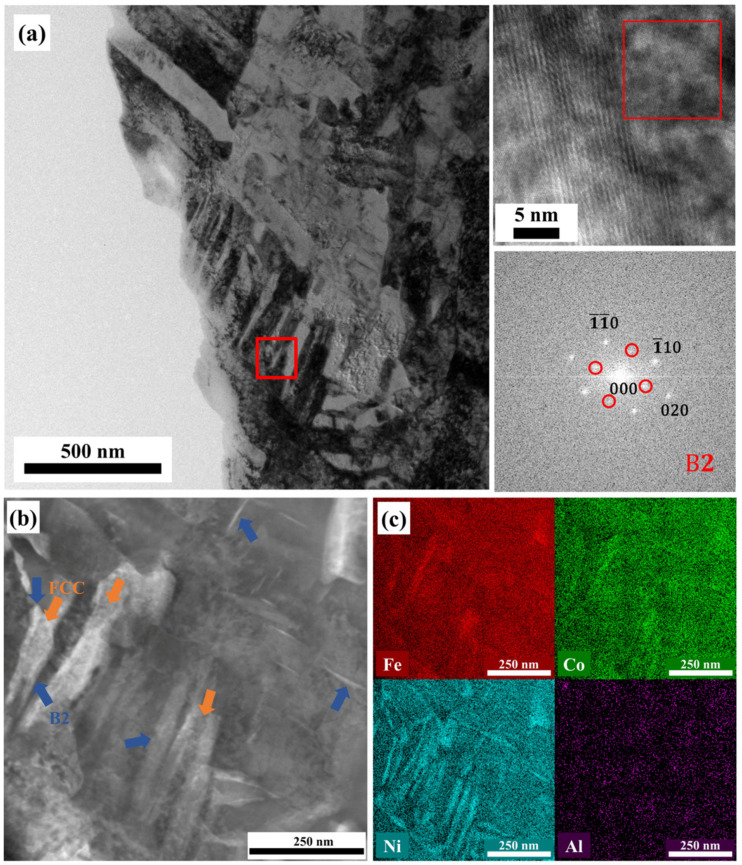
The microstructure of AlCoFeNi after aging at 450 °C for 4h. (**a**) Bright-field TEM image and HRTEM image, (**b**) microstructure of precipitates with (**c**) TEM-EDS map for the elemental distribution of Fe, Co, Ni, and Al, respectively.

As shown in Figure 10, a TEM analysis was carried out to further observe the microstructure of AlCoFeNi after aging at 450 °C for 4h. Figure 10a shows a bright-field TEM image and an HRTEM image. The BCC and B2 phases can be seen in this figure, but it is difficult to detect the FCC phase. Figure 10b shows the detailed microstructure of the precipitates, and Figure 10c shows the elemental distribution of Fe, Co, Ni, and Al, respectively. Two distinct precipitates can be seen in Figure 10c: one is enriched with Fe and Co, and the other is enriched with Ni and Al. This can be compared with the results of [23], which include B2 grain-enriched Ni and Al and the FCC grain-enriched Fe and Co. Therefore, the phase pointed by the blue arrows may be the B2 phase, while the phase pointed by the orange arrows may be the FCC phase, as shown in the Figure 10b. The amount of the B2 grain is more than the amount of the FCC grain. Some B2 grains are at the boundary of the FCC grain, and some small acicular B2 grains are in the matrix (Figure 10c). Overall, the SLM AlCoFeNi MEA changed from a single BCC structure to a polyphase structure of BCC + B2 + FCC after aging at 450 °C for 4 h.

### 3.3. Mechanical Properties

The Rockwell hardness values are outlined in Table 2. Initially, the as-built samples exhibited a hardness of 32.5 HRC. A significant improvement in hardness was detected for the aged samples. After aging for 2 h at a temperature of 410 °C, 430 °C, and 450 °C, the hardness was recorded as 41.4 HRC, 44.3 HRC, and 44.1 HRC, respectively. Within the extent of the present experiment, specifically from 430 °C to 450 °C, the alloy displayed its optimum aging hardness. Subsequently, after aging at 450 °C for 2 h, 4 h, and 6 h, the corresponding hardness was 44.1 HRC, 45.7 HRC, and 46.1 HRC, respectively. This trend illustrated that the hardness progressively improved with longer aging durations in the present experiment.

**Figure 11:**
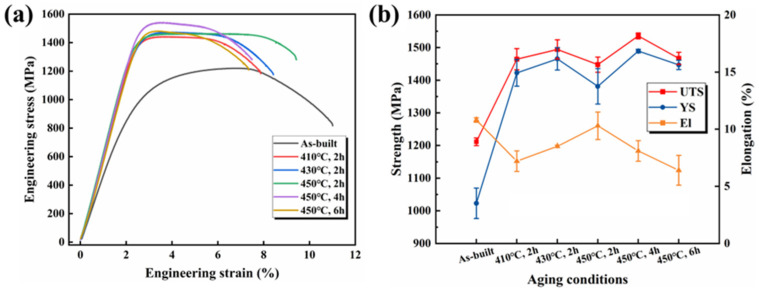
(**a**) Engineering stress–strain curves of the as-built and heat-treated AlCoFeNi MEA, and (**b**) the UTS, YS, and El variations of these samples.

The engineering stress–strain curves for both the as-built and heat-treated samples are shown in Figure 11a and are summarized in Table 3. Figure 11b illustrates the strength and toughness variations of these samples. Notably, the as-built samples exhibited a YS and UTS of around 1023 MPa and 1211 MPa, respectively, with the highest El (10.8%). Both the YS and UTS of the aged samples exhibited significant improvements, although little variation could be detected, which was attributed to the different aging temperatures. The UTS values for the samples aged at 410 °C, 430 °C, and 450 °C for 2 h were 1464 MPa, 1494 MPa, and 1447 MPa, respectively. The El values for the aged samples were lower compared with the as-built samples and exhibited a marginal improvement with an increase in aging temperature. For the samples aged for 2 h at 410 °C, 430 °C, and 450 °C, El values of 7.2%, 8.5%, and 10.3% were detected, respectively. After aging for 2 h, 4 h, and 6 h at 450 °C, a UTS of 1447 MPa, 1535 MPa and 1466 MPa, a YS of 1381 MPa, 1489 MPa, and 1446 MPa, and an El of 10.3%, 8.1%, and 6.4%, respectively, were observed. With an increasing aging duration, the El of the samples aged at 450 °C gradually decreased.

**Figure 12:**
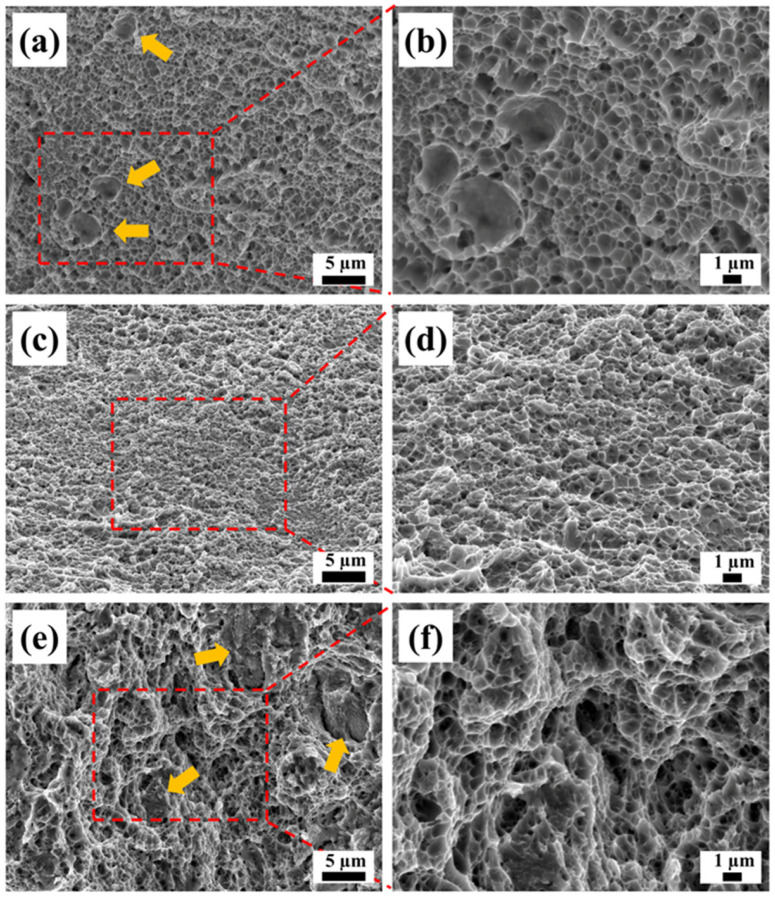
Fracture morphology of the tensile tested samples: (**a**,**b**) as-built (the arrows point to prominent dimples), (**c**,**d**) AT at 450 °C for 2 h, and (**e**,**f**) AT at 450 °C for 6 h (the arrows point to cleavage structures).

Figure 12 presents the typical fracture morphology of the tensile-tested samples. As shown in Figure 12a,b, the fracture of the as-built samples exhibited some prominent dimples, along with a plentiful distribution of uniformly dispersed small dimples. These features indicated a typical ductile fracture characteristic. Following aging at 450 °C for 2 h, the fracture surface exhibited numerous uniformly distributed small dimples, indicating typical ductile fracture characteristics. Subsequently, after 6 h of aging at 450 °C, some cleavage structures and a few small dimples could be observed on the fracture surface, which is a sign of a mixed fracture involving both toughness and brittleness.

## 4. Discussion

### 4.1. Changes in the Microstructure

The as-built samples predominantly consisted of elongated columnar grains and fine equiaxed grains. During the SLM process, the temperature gradient in the direction of build extension acted as the driving force for the growth of columnar grains. Consequently, columnar grains grew perpendicularly to the boundary of the melt pool, resulting in cellular crystal structures within the melt pool, which are typical structures of SLMs [21]. The rapid cooling rate of the molten pool in the SLM process was in the range of approximately 10^3^ to 10^8^ K/s, which led to a finer grain in the as-built samples compared with the same alloy prepared by conventional methods [33]. The preferred orientation in the <101> direction of the as-built samples may be related to the temperature gradient caused by the laser moving trajectory. The temperature gradient has a certain effect on the growth of the grain [34].

As mentioned above, the alloy powder underwent a process of high temperature melting, rapid solidification, and a complex thermal cycle process in the SLM process. These led to the formation of a large number of martensitic structures in the SLM AlCoFeNi MEA after the aging treatment [35], as shown in Figure 7. With the increase in aging temperature and duration, the martensitic structures decreased gradually and dissolved into the matrix. Compared with the as-built samples, it can be seen in Figure 9a,c,e that the number of fine grains increased gradually after the aging treatment. This can be attributed with the precipitated phase during aging, and by comparing the IPF maps with the phase maps, it can be seen that some fine grains can correspond to the precipitated FCC phase. With the increase in aging temperature and duration, the nucleation rate of the precipitated phase increased, and the nano-sized precipitates grew gradually. Meanwhile, the disappearance of the original grain structures was also closely related to the formation of the precipitated phase. The aging treatment made the microstructure and precipitate distribution of the alloy more uniform.

### 4.2. Precipitated Phases

The Thermo-Calc software was employed to calculate the phase compositions of the Al_0.3_CoFeNi MEA, which were proven to be valid [11]. In line with this approach, the TCHEA3 package within the Thermo-Calc 2022b software was used to estimate the phase fractions in the present AlCoFeNi MEA. The computational results successfully distinguished the FCC and L1_2_ phases; however, it was difficult to distinguish the BCC and B2 phases. Drawing from both the experimental and computational results, the phase composition of the AlCoFeNi MEA was temperature-dependent, as shown in Figure 12, and parts of the two dotted lines were the aging temperature intervals for this experiment. When the temperature increased from 410 to 450 °C, there was a noticeable decrease in the content of the BCC/B2 phase, accompanied by a corresponding increase in the content of the FCC phase, which was consistent with the experimental results. It should be noted that there is a high content of the L1_2_ phase in the calculation results, but no globular L1_2_ phase [36] is found in the experimental results. This may be due to the fact that the addition of small amounts of Al may form little or no L1_2_ phase. Another reason for this is the inaccuracies in solution thermodynamic modeling [23]. However, the results of the calculations and experiments show the same variation in FCC precipitates with an increase in aging temperature.

As mentioned above, the introduction of Al promoted the ordering of the alloy. In the case of the as-built sample, it predominantly exhibited a single BCC phase. This can be attributed to the extremely rapid cooling of the molten pool during the SLM process. Consequently, no other phases precipitated. However, during aging, a significant change occurred due to the relatively low nucleation barrier potential between the BCC and B2 phases [11]. Meanwhile, there is a strong tendency for Al and Ni to form precipitates [37]. A B2 phase containing a higher concentration of Ni and Al may preferentially grow within the BCC matrix [10]. In contrast, the nucleation barrier potential between the BCC and FCC phases are significantly higher. Consequently, the amount of the FCC phase is less. Meanwhile, Fe + Co enrichment (and Ni + Al depletion) in the FCC phase [23] resulted in some B2 grains located at FCC grain boundaries.

### 4.3. Strengthening Mechanisms

**Figure 13:**
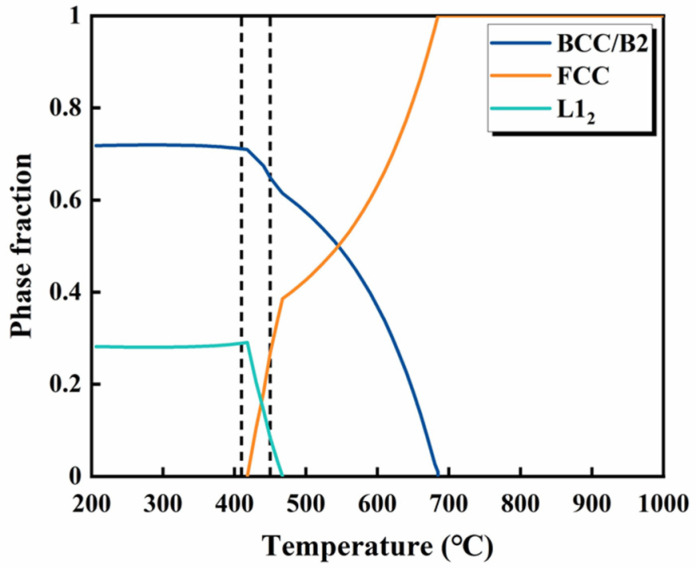
Phase fraction vs. temperature plot for AlCoFeNi. The two dash lines represent the aging temperature range in this study.

The hardness values and engineering stress–strain curves of the samples after different heat treatments are illustrated in Table 2 and Figure 11. Compared with the as-built samples, the aged samples show higher strength and hardness values. The as-built samples showed the original heterogeneous cellular structure and the single-phase solid solution without precipitates, resulting in limited strengthening effects. However, as shown in Figure 9, after aging, the number of fine grains increased. With an increase in aging temperature and duration, the number and size of the fine grains increased gradually, and a higher volume fraction of the grain boundaries was observed. This resulted in fine-grain strengthening and grain-boundary strengthening, which can be synthesized and expressed by Hall–Petch strengthening [21]. Meanwhile, precipitation strengthening had a great influence on the mechanical properties of the aged samples. The aging process led to FCC and B2 phases, and these precipitations contributed to hindering the grain boundary migration, which can effectively improve the strength and hardness of alloys. As shown in Figure 6 and Figure 13, the fraction of the FCC phase gradually increased with an increase in aging temperature and duration. This indicates an enhancement in precipitation strengthening. In addition, solid solution strengthening, which was caused by lattice distortion, which was caused by the Al, contributes to the strength and hardness of alloys [38]. Overall, the improvement in the mechanical properties of the aging AlCoFeNi MEA is attributed to the combined effect of multiple strengthening mechanisms of solid solution strengthening, precipitation strengthening, and Hall–Petch strengthening, rather than a simple superposition of several strengthening mechanisms.

Notably, the El gradually increased when aging at 410 °C, 430 °C, and 450 °C for 2 h, which is closely related to the formation of a great deal of FCC phases. This was significantly higher than that of the samples aged at other aging temperatures. When aged at 450 °C, more FCC precipitates were observed, but the El gradually decreased with an increase in duration. This may be because a more uniform distribution of precipitates leads to an increase in hardness, which is accompanied by a decrease in toughness. After aging at 450 °C for 4 h, the hardness, UTS, YS, and El were around 45.7 HRC, 1535 MPa, 1489 Mpa, and 8.5%, respectively. This resulted in a well-balanced alloy with high strength, hardness, and toughness. Overall, these results reflect the complex interaction of multiple strengthening mechanisms significantly enhancing the mechanical properties of the AlCoFeNi MEA.

## 5. Conclusions

In this study, a novel three-phase BCC + B2 + FCC AlCoFeNi MEA was designed and manufactured using the SLM method. The microstructure evolution and mechanical properties before and after heat treatment were investigated. The key findings are summarized as follows:

(1) SLM can be used to fabricate an AlCoFeNi MEA, which shows good performance. The as-built samples exhibit a single BCC phase consisting of columnar and equiaxed grains, which achieved a high density of 99.97%, a hardness of 32.5 HRC, a YS of 1023.1 MPa, a UTS of 1211.5 MPa, and an El of 10.8%.
(2) Phase transformation occurred during the aging treatment, and a complex three-phase structure comprising BCC, B2, and FCC phases was achieved. With an increase in aging temperature and duration, an evident reduction in the content of the BCC/B2 phases was detected, accompanied by an increase in the FCC phases. It is obvious that the influence of the aging temperature on phase transformation is more significant than the aging time.
(3) The MEA achieved a well-balanced combination of hardness, strength, and toughness after the heat treatment. Following aging at 450 °C for 4 h, it exhibited impressive mechanical properties, including a hardness of 45.7 HRC, a UTS of 1535.7 MPa, a YS of 1489.3 MPa, and an El of 8.1%.
(4) An appropriate aging temperature duration can significantly improve the mechanical properties of alloys. Hall–Petch strengthening, solid solution strengthening, and precipitation strengthening play an important role in this process.

## Figures and Tables

**Table 1:** Heat treatment parameters.

Condition	Heat Treatment Process
A	No treatment, as-built via SLM
B	Aging treatment (AT) at 410 °C for 2 h
C	AT at 430 °C for 2 h
D	AT at 450 °C for 2 h
E	AT at 450 °C for 4 h
F	AT at 450 °C for 6 h

**Table 2:** Rockwell hardness of the as-built and heat-treated AlCoFeNi MEA.

Conditions	Hardness (HRC)
As-built	32.5 ± 0.9
AT at 410 °C for 2 h	41.4 ± 0.3
AT at 430 °C for 2 h	44.3 ± 0.5
AT at 450 °C for 2 h	44.1 ± 0.2
AT at 450 °C for 4 h	45.7 ± 0.1
AT at 450 °C for 6 h	46.1 ± 0.1

**Table 3:** Tensile properties of the as-built and heat-treated AlCoFeNi MEA.

Conditions	Ultimate Tensile Strength (MPa)	Yield Stress (MPa)	Elongation (%)
As-built	1211 ± 11.8	1023 ± 46.5	10.8 ± 0.2
AT at 410 °C for 2 h	1464 ± 32.5	1422 ± 41.1	7.2 ± 0.9
AT at 430 °C for 2 h	1494 ± 29.4	1465 ± 34.1	8.5 ± 0.1
AT at 450 °C for 2 h	1447 ± 23.1	1381 ± 54.3	10.3 ± 1.2
AT at 450 °C for 4 h	1535 ± 9.1	1489 ± 5.65	8.1 ± 0.9
AT at 450 °C for 6 h	1466 ± 18.8	1446 ± 14.3	6.4 ± 1.3

## Data Availability

Data are available on request from the authors.
